# Supplementary material for: Listen or Read? The Impact of Proficiency and Visual Complexity on Learners’ Reliance on Captions
Source: Behav Sci (Basel). 2025 Apr 17;15(4):542. doi: 10.3390/bs15040542 (PMC12024247; doi:10.3390/bs15040542)
Supplement: Supplementary file 1 [file behavsci-15-00542-s001.zip › behavsci-3505320-supplementary.pdf]

Studies investigating how language learners process captioned videos

Studies	Participants	Video(s) used	Tools	Main findings
**Cross-sectional studies (Lower-proficiency learners)**				
Caimi (2006)	15 Italian pre-intermediate EFL learners	Not specified	Interview	Mainly read captions: Reading, viewing and listening impeded some learners' comprehension
Sydorenko (2010)	26 second-semester US university Russian FL learners	Three 2–3 min Russian comedy clips	Questionnaire	(1) Mainly read captions (2) They paid most attention to captions, then to video, then to audio (3) They consider video to be the most helpful, captions the least; (4) some participants have difficulty attending to all three modalities
Winke et al. (2013)	33 second-year college-level foreign language (Arabic, Chinese, Russian, Spanish) learners	Two 3-5 mins English - documentaries about salmon and bears, with a single narrator.	Eye tracking, Interview	Learners mainly read captions, fixing on the captions area 68% of the time that the captions were present on screen.
**Cross-sectional studies (Mixed lower/higher-proficiency learners)**				
Pujol`a (2002)	22 upper-intermediate adult Spanish EFL learners	News items presented in different formats	Screen video-recording, observation, verbal report, Interview	(1) Less-proficient learners relied more on captions, using captions as a scaffold (2) Proficient learners used captions less often, just to back up their comprehension when needed
Taylor (2005)	35 US university Spanish FL (17 first-year, 18 third and fourth-year)	A segment titled "Ricos sabores" from the videotape of the first - year Spanish textbook Puentes.	Learner reflective written protocols	(1) 35% (6 of 17) first-year and 11% of 3-year learners found captions distracting and had difficulty using spoken, visual and caption cues together (2) Most learners

				(especially experienced leaners) used three channels simultaneously<br>(3) Several students ignored the sound altogether
**Cross-sectional studies (higher-proficiency learners)**				
Chai and Erlam (2008)	10 Chinese upper-intermediate ESL learners in Auckland	A 10 - minute scene selected from the DVD movie *Howl's Moving Castle*	A semi - structured interview	Mainly read captions
**Longitudinal studies**				
Vanderplank (1988)	15 upper-intermediate to advanced European ESL in UK<br><br>8 low-intermediate to advanced Arabic students as the study group	authentic TV programs (police series, a popular soap and a weekly holiday report program	Learner reflective written protocols	(1) Two learners were distracted from the audio by captions<br>(2) European students found captions not distracting<br>(3) Arabic students thought the captions changed too fast<br>(4) Some learners alternated use of spoken, caption cues as needed<br>(5) Some learners simultaneously used spoken, visual, caption cues
Vanderplank (2016)	18 mainly upper-intermediate to advanced UK university; Various European FLs	Movies of various genres	Learner diary; Questionnaire; Interview	(1) Less-proficient learners used captions as a scaffold<br>(2) Proficient learners used captions to back up their comprehension when needed
Vanderplank (2019)	36 intermediate level and above learners of French,	Movies of various genres	Hard-copy and online diary, questionnaire, feedback checklist	(1) For most participants, their viewing behavior often changed during a film.<br>(2) Participants tended to use captions more at the

	German, Italian and Spanish (18 for the first trial and 18 for the second trial)			start of viewing, when faced with challenging material above their proficiency level.<br>(2) They were able to follow and understand less-challenging films well without captions.
